# Low-Cost and Rapid Production of Calcium Formate from Cockle Shell Waste for Sustainable Waste Recycling

**DOI:** 10.3390/ijms27083520

**Published:** 2026-04-15

**Authors:** Chaowared Seangarun, Banjong Boonchom, Somkiat Seesanong, Wimonmat Boonmee, Sirichet Punthipayanon, Nongnuch Laohavisuti, Pesak Rungrojchaipon

**Affiliations:** 1 Material Science for Environmental Sustainability Research Unit, School of Science, King Mongkut’s Institute of Technology Ladkrabang, Bangkok 10520, Thailand; chaowared@gmail.com (C.S.); wimonmat.bo@kmitl.ac.th (W.B.); sirichet@g.swu.ac.th (S.P.); pesak.ru@kmitl.ac.th (P.R.); 2 Department of Chemistry, School of Science, King Mongkut’s Institute of Technology Ladkrabang, Bangkok 10520, Thailand; 3 Municipal Waste and Wastewater Management Learning Center, School of Science, King Mongkut’s Institute of Technology Ladkrabang, Bangkok 10520, Thailand; 4 Office of Administrative Interdisciplinary Program on Agricultural Technology, School of Agricultural Technology, King Mongkut’s Institute of Technology Ladkrabang, Bangkok 10520, Thailand; somkiat.se@kmitl.ac.th; 5 Department of Biology, School of Science, King Mongkut’s Institute of Technology Ladkrabang, Bangkok 10520, Thailand; 6 Department of Sports Science, Faculty of Physical Education, Srinakharinwirot University, Bangkok 10110, Thailand; 7 Thailand Association of Mixed Martial Arts, 431/19 Rajchadapisek Road, Yannawa, Chongnonsee, Bangkok 10120, Thailand

**Keywords:** calcium formate, biological calcium carbonate, cockle shell waste, renewable calcium source

## Abstract

Calcium formate (Ca(HCOO)_2_) is an important industrial chemical widely used in construction, feed additives, and various chemical processes. In this work, calcium formate was synthesized from cockle shell waste and concentrated formic acid (50%, 60%, and 70% *w*/*w*) by a simple, rapid, low-cost, and environmentally friendly process, denoted as CF50, CF60, and CF70, respectively. The chemical and physical properties of as-synthesized calcium formate using cockle shells as a renewable calcium source were investigated by Fourier transform infrared (FT-IR), X-ray diffraction (XRD), X-ray fluorescence (XRF), Thermal gravimetric analysis (TGA), and scanning electron microscopy (SEM) techniques. The FTIR and XRD results revealed that the samples prepared using 50% and 60% formic acid produced well-crystallized α-calcium formate. In contrast, the reaction using 70% formic acid generated a strongly exothermic reaction, which hindered the complete conversion of calcium carbonate and resulted in the presence of residual CaCO_3_ in the final product. Similarly, the SEM images of the CF50 and CF60 samples show the slick surface of orthorhombic crystals of calcium formate; on the other hand, the SEM image of CF70 shows some small particles of aragonite on the surface of the calcium formate crystals. The 60% formic acid provided the optimal synthesis condition, yielding pure calcium formate with the shortest synthesis time. Overall, the proposed approach provides a simple, rapid, and cost-effective route for producing calcium formate from shell waste. Furthermore, the utilization of cockle shell waste as a renewable calcium source contributes to waste valorization, reduces environmental impacts associated with shell disposal, and minimizes dependence on mined limestone resources, supporting sustainable resource utilization within a circular economy.

## 1. Introduction

Recently, interest in waste recycling has been increasing for both the sustainability of human society and environmental protection [1]. The growing scarcity of natural resources has become a major problem for both developed and developing countries. As a result, increasing attention has been focused on environmentally friendly approaches that promote waste recycling and reuse. Consequently, researchers have explored various green technologies and innovative methods to convert waste materials into valuable products [2]. The rapid growth of global aquaculture and fisheries has become a significant contributor to environmental problems, generating large amounts of calcareous biowaste, primarily discarded mollusk shells, which often accumulate in landfills or coastal areas without proper management [3]. The cockle is a native mollusk widely found in coastal regions of Southeast Asia, particularly in Malaysia, Thailand, and Indonesia [4]. Annually, the industrial production of cockle shells from fishery waste exceeds 0.4 million tons [5]. The processing of cockle shells also increases significant amounts of shell waste, and their final destination is an environmental problem [6]. Therefore, the recycling and valorization of cockle shell waste have attracted growing attention as promising strategies for resource recovery and environmental protection. Developing sustainable technologies to convert cockle shell waste into high-value products can contribute to resource-efficient utilization, waste minimization, and the advancement of a circular economy based on marine biowaste [7].

Cockle shells are mainly composed of calcium carbonate (CaCO_3_), typically accounting for approximately 98–99% of their total composition, with minor amounts of organic matrix and trace minerals [8]. This high calcium carbonate content makes cockle shells an excellent renewable calcium source for the synthesis of various calcium-based materials. Previous studies have reported the successful conversion of shell-derived CaCO_3_ into a variety of value-added calcium compounds, including calcium oxide [9], calcium acetate [10], calcium lactate [11], calcium formate [12], calcium citrate [13], calcium pyrophosphate [14], and hydroxyapatite [15]. Among these compounds, calcium formate (Ca(HCOO)_2_) is an important industrial chemical widely used in several applications, including concrete accelerating agents [16,17,18], feed additives [19], leather tanning [20], textile processing [21], and de-icing agents [22]. Its global production exceeds 100,000 tons per year and is expected to continue growing due to its strong potential for generating a wide range of C1 chemicals [23].

Calcium formate (Ca(HCOO)_2_) is a calcium salt that exhibits four different modifications (α, β, γ and δ). The α-Ca(HCOO)_2_ is known as a synthetic phase of calcium salt. Calcium formate can be synthesized from a reaction between calcium carbonate (CaCO_3_) and formic acid (HCOOH) [24]. The structure of α-Ca(HCOO)_2_ is more stable than other structures at room temperature [25]. Currently, calcium formate is industrially produced from limestone, i.e., CaCO_3_ derived from calcite and dolomite minerals, through a two-step process. This process involves the calcination of CaCO_3_ to produce CaO or Ca(OH)_2_ with the release of CO_2_, followed by either the Cannizzaro reaction or carbonylation with aldehydes (R–CHO) to form calcium formate [23]. In addition, calcium formate was prepared from the reaction between calcium hydroxide and formaldehyde solution as well as the reaction of soluble calcium salts, such as calcium chloride and calcium nitrate, with salts of formic acid, such as sodium formate, which results in the formation of two other salts as final products as a result of an exchange reaction [26]. Recently, the utilization of biogenic calcium carbonate as a precursor for calcium formate synthesis has attracted increasing research interest. For instance, Wang et al. [12] reported the controlled synthesis of calcium formate using biological aragonite derived from shell materials. In their approach, the shell-based CaCO_3_ was reacted with dilute formic acid to dissolve calcium. After that, ethyl alcohol was used as an anti-solvent to induce crystallization and recovery of calcium formate crystals with controlled morphology. Although this approach enables controlled crystal formation, the use of organic solvents and additional precipitation steps may increase process complexity, chemical consumption, and production costs. To address these limitations, the present study proposes a simpler and more cost-effective method for producing calcium formate from cockle shell waste. High-concentration formic acid solutions were directly reacted with finely ground cockle shell powder, allowing rapid formation of calcium formate without the use of additional solvents for precipitation. This approach simplifies the synthesis process, reduces chemical usage, and potentially lowers production costs while promoting the valorization of shell waste.

This research focuses on identifying the optimum synthesis conditions by reacting cockle shell-derived CaCO_3_ with varying concentrations of formic acid (50%, 60%, and 70%) and analyzing the chemical and physical properties of the synthesized products. This work investigates how acid concentration influences the reaction time, yield, solubility, chemical properties, and physical properties of the resulting α-(Ca(HCOO)_2_). Therefore, the proposed process offers several direct advantages, including shorter reaction time, simplified processing steps, reduced chemical consumption, and lower production costs compared with previously reported methods. In addition, the solvent-free precipitation approach improves the environmental friendliness of the synthesis process. Beyond these direct benefits, using cockle shell waste as a renewable calcium source offers significant environmental advantages by reducing shell waste disposal and minimizing reliance on limestone, the primary raw material in current industrial production processes. This strategy not only promotes waste valorization and sustainable resource utilization but also contributes to the development of environmentally responsible chemical processes aligned with circular economy principles.

## 2. Results and Discussion

### 2.1. Fourier Transform Infrared (FTIR)

**Figure 1:**
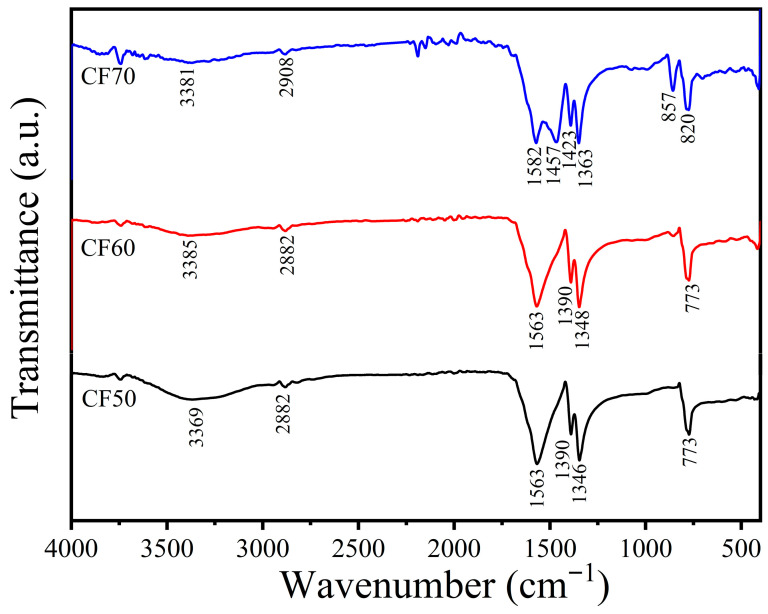
Fourier transform infrared (FTIR) spectra of all the synthesized calcium formate (Ca(HCOO)_2_); CF50, CF60, and CF70 samples.

The fundamental mode bands in the FTIR spectra of the CF50, CF60, and CF70 samples are shown in Figure 1, and the fundamental band assignments are given in Table 1. The characteristic peaks of HCOO^−^ anion were found in the spectra of the CF50, CF60, and CF70 samples. The weak band of C–H asymmetric stretching (*ν*_as_) is observed at around 2908–2842 cm^−1^. The strong band at 1682 cm^−1^ is attributed to the symmetric stretching (*ν*_s_) of C=O in the carbonyl group. The characteristic bands of C–O asymmetric stretching (*ν*_as_) were observed at 1363 cm^−1^. The vibrational peaks at 1423 and 820 cm^−1^ are assigned to the bending (*δ*) vibration of O–C–H and O=C–O, respectively. The characteristic peaks of HCOO^−^ anion in this work corresponded to the vibrational spectra of α-Ca(HCOO)_2_ found from a natural source, which was reported in the previous research [27]. The weak broad bands appeared at around 3711–3029 cm^−1^ and are related to O–H stretching of moisture (H_2_O). Additionally, the infrared spectrum of CF70 shows characteristic absorption peaks of CO_3_^2−^ anions, corresponding to the C-O asymmetric stretching (ν_as_) and O-C-O out-of-plane bending (*δ*), observed at 1457 and 857 cm^−1^, respectively [28,29].

### 2.2. X-Ray Diffraction

**Figure 2:**
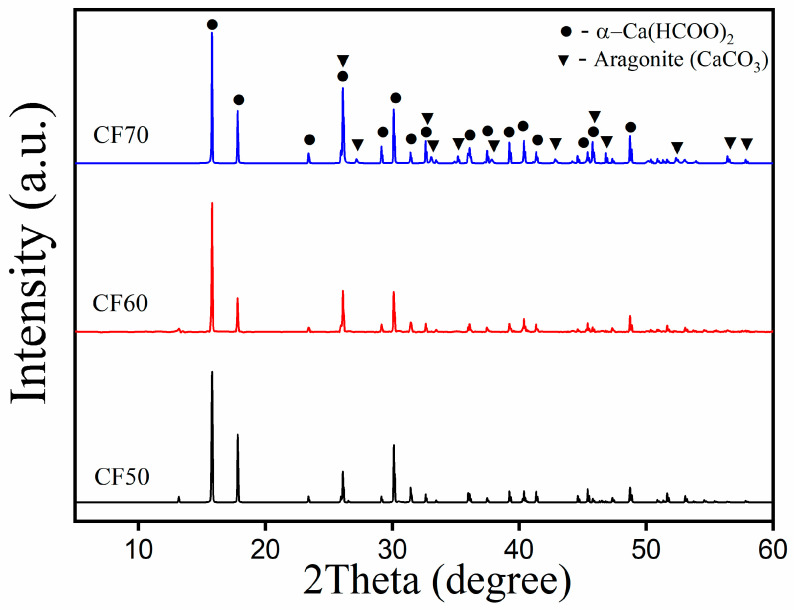
X-ray diffraction patterns of all the synthesized calcium formate (Ca(HCOO)_2_); CF50, CF60, and CF70 samples.

Calcium formate is generally reported to exhibit four different polymorphs: α, β, γ, and δ [30]. To identify the phase of the synthesized calcium formate crystal from this work, the X-ray diffractometer (XRD) was used to analyze the XRD patterns of the CF50, CF60, and CF70 samples, which are shown in Figure 2. The resulting diffraction patterns were further analyzed by comparing them with reference data from international diffraction databases to confirm the chemical composition [31]. The diffraction patterns of CF50 and CF60 exhibit only the characteristic peaks of orthorhombic α-Ca(HCOO)_2_, corresponding to the standard data in PDF Card No. 01-074-6923. The XRD patterns of the synthesized calcium formate from this work were similar to those of calcium formate found in natural sources [27,32], whereas the XRD pattern of CF70 indicates incomplete conversion, as evidenced by the presence of diffraction peaks corresponding to both orthorhombic α-Ca(HCOO)_2_ (PDF card No.01-074-6923) and the residual aragonite phase of CaCO_3_ (PDF card No.01-080-2786). The XRD pattern of the CF70 sample is consistent with the FTIR results, which revealed the presence of carbonate (CO_3_^2−^) functional groups.

The FTIR and XRD results of CF70 clearly confirm the presence of residual calcium carbonate in the obtained product. This phenomenon can be attributed to the exothermic reaction between formic acid and CaCO_3_. Upon rapid addition of formic acid, the acid–carbonate reaction proceeds vigorously, releasing a significant amount of heat very quickly. The sudden temperature increase may promote partial evaporation of formic acid, given its relatively high volatility in aqueous solutions. Consequently, a fraction of the added acid may be lost through evaporation before fully reacting with CaCO_3_. This reduction in the effective amount of available acid can limit the complete conversion of CaCO_3_ to calcium formate, resulting in the detection of unreacted CaCO_3_ in the final CF70 product. A similar effect has been reported by Seesanong et al. [33], where the use of highly concentrated phosphoric acid (70% *w*/*w*) for the reaction with oyster shell-derived CaCO_3_ generated a strong exothermic reaction, leading to elevated temperatures that partially promoted the formation of dicalcium phosphate (CaHPO_4_) instead of the desired monocalcium phosphate (Ca(H_2_PO_4_)_2_).

### 2.3. Thermal Decomposition

**Figure 3:**
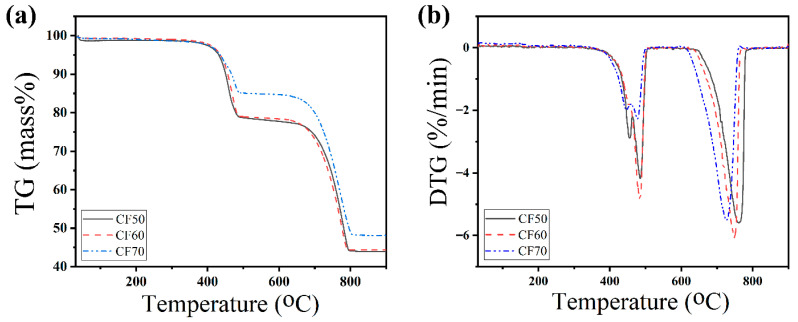
TG (**a**) and DTG (**b**) curves of all the synthesized calcium formate (Ca(HCOO)_2_); CF50, CF60, and CF70 samples.

Thermal analysis was conducted to evaluate the thermal decomposition behavior of the synthesized calcium formate (Ca(HCOO)_2_). TG and DTG curves of the CF50, CF60, and CF70 samples are shown in Figure 3. In the TG curves of all synthesized calcium formate, two distinct mass-loss steps can be observed. The first decomposition step of calcium formate samples occurred between approximately 400 °C and 500 °C, as indicated by the DTG curves. The first mass-loss step corresponded to the decomposition of calcium formate, which produced calcium carbonate and formaldehyde, as described by Equation (1) [34,35]. The first thermal decomposition step of the CF50 and CF60 samples shows a measured mass loss of about 21.10% wt, which is close to the theoretical mass loss (calculated mass loss: 23.08% wt). However, the first mass-loss step of the CF70 sample shows a measured mass loss of 14.64% wt, which is significantly lower than that of the CF50 and CF60 samples.

First decomposition step:

Ca(HCOO)_2_(s) → CaCO_3_(s) + HCHO(g) (1)

The second decomposition step was observed approximately between 650 °C and 800 °C. This step was associated with the thermal decomposition of calcium carbonate (CaCO_3_), resulting in the formation of calcium oxide (CaO) and the release of carbon dioxide (CO_2_) according to Equation (2) [34,35]. The second thermal decomposition step of the CF50 and CF60 samples shows a measured mass loss of about 34.67%wt, which is close to the theoretical mass loss (calculated mass loss: 33.85%wt). In contrast to the results of the first mass-loss step, the second mass-loss step of the CF70 sample shows the measured mass loss of 37.12%wt, which is significantly higher than the thermal decomposition of the CF50 and CF60 samples. The thermal decomposition profile of the synthesized calcium formate derived from cockle shells is consistent with previously reported decomposition patterns of calcium formate [34,35,36], confirming that the synthesized product exhibits typical thermal degradation behavior.

Second decomposition step:

CaCO_3_(s) → CaO(s) + CO_2_(g) (2)

In the first decomposition step, the CF70 sample exhibited a lower mass loss (~6.46%) than the CF50 and CF60 samples, due to the presence of unreacted calcium carbonate from incomplete reaction during synthesis, consistent with the FTIR and XRD results. In contrast, the mass loss of the CF70 sample in the second step was higher than that of the other samples (~2.45%). This result is due to the thermal decomposition, which involves both the residual CaCO_3_ originally present in the sample and the CaCO_3_ formed from the thermal decomposition of calcium formate.

### 2.4. Chemical Composition

An X-ray fluorescence spectrophotometer was used to determine the chemical compositions of the synthesized calcium formate. The main composition and impurities of CF50, CF60, and CF70 are shown in Table 2. All prepared samples show a high calcium oxide (CaO) content, exceeding 93% by weight. CF50 and CF60 show the highest calcium content at 95.2–95.5% CaO. Additionally, there are 4.50–4.80% impurities, including Na_2_O, MgO, Al_2_O_3_, SiO_2_, P_2_O_5_, SO_3_, Cl, K_2_O, TiO_2_, MnO, Fe_2_O_3_, and SrO, which may originate from the raw materials [36]. These impurities were also detected in the XRF results of raw cockle shell powders reported by Awang-Hazmi et al. [4]. The purity of the synthesized calcium compound was highest at a high acid concentration, but only up to a point; beyond that, the concentration became too high, triggering a strong exothermic reaction that caused evaporation of formic acid. This result was in line with previous works that reported the synthesis of calcium phosphate [33], calcium acetate [29], and calcium lactate [37] using highly concentrated acid.

### 2.5. Morphologies of Samples

**Figure 4:**
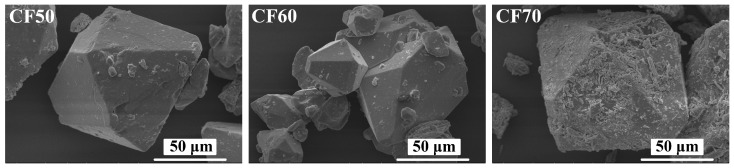
Scanning electron microscopic (SEM) images of the synthesized calcium formate (Ca(HCOO)_2_).

Morphologies of synthesized calcium formate (Ca(HCOO)_2_) were observed using a Leo SEM 400 scanning electron microscope (SEM) at 2500× magnification, with gold coating. As demonstrated in Figure 4, SEM images of CF50 and CF60 show smooth surfaces of orthorhombic calcium formate crystals with sizes ranging from 20 to 100 μm. The morphologies of synthesized calcium formate from cockle shell powders were similar to natural α-calcium formate, which was found in sediments of Alkali Lake, Oregon, USA [27] with a smaller particle size. In addition, the SEM image of the CF70 sample shows numerous rod-like particles consistent with the morphology of cockle shell-derived CaCO_3_, as reported in a previous study [38]. This observation is consistent with the FTIR, XRD, and TGA results, supporting the incomplete conversion and indicating formate ion deficiency during the CF70 synthesis due to the rapid evaporation of formic acid under strongly exothermic conditions.

### 2.6. Production Results

Calcium formate (Ca(HCOO)_2_) samples (CF50, CF60, and CF70) were prepared from cockle shell powders (CaCO_3_) and concentrated formic acid (50%, 60%, and 70% *w*/*w*) according to the reaction in Equation (3). The reaction temperature, drying time, yield, and soluble percentage of all prepared products, which were affected by various concentrations of formic acid, are shown in Table 3. All experiments were performed in triplicate, and the results are reported as average values with standard deviations. The reported temperatures correspond to the maximum temperatures reached during the reaction due to the exothermic reaction without external heating. The CF50 and CF60 samples obtained yields of 95.88% and 96.54% at a reaction temperature under 50 °C using a reaction time of 15–20 min. However, the reaction with the CF70 sample ended in less than 1 min, and the yield decreased to 88.69%. The soluble fraction percentages of the prepared calcium formate samples showed a similar trend to the yield results. The CF50 and CF60 samples exhibited high soluble fractions of 93.87% and 94.49%, respectively. In contrast, the CF70 sample showed a significantly lower soluble fraction of 63.14%, confirming the evaporation of formic acid due to the strong exothermic reaction, as discussed in the previous sections.

Based on the experimental results, the concentration of formic acid plays a crucial role in determining reaction behavior, conversion efficiency, and product quality in the synthesis of calcium formate from cockle shell-derived CaCO_3_. Among the investigated conditions, 60 wt% formic acid provided the optimal synthesis condition, yielding the highest yield, the largest soluble fraction, and the shortest reaction time, while enabling complete conversion of CaCO_3_ to calcium formate. At this concentration, the reaction proceeded under moderate exothermic conditions, allowing sufficient contact time between the reactants and enabling complete conversion of CaCO_3_ to α-calcium formate within a short reaction time (15 min). In contrast, the reaction with 70% formic acid became excessively exothermic, leading to rapid acid evaporation and a deficiency of formate anions in the reaction system. This phenomenon led to incomplete conversion, leaving residual CaCO_3_ in the final product and significantly reducing both solubility and product yield. Deficiency of anions caused by a very highly exothermic reaction due to the usage of a high concentration of acid (70% *w*/*w*) was also observed in previous works that prepared calcium phosphate [33], calcium acetate [29], and calcium lactate [37] from shell wastes using various types of highly concentrated acid.

The economic feasibility of the proposed synthesis route is an important factor for evaluating its potential for industrial-scale application. In this work, the cost of producing calcium formate from cockle shell-derived calcium carbonate and formic acid was estimated based on the optimal reaction condition (60% *w*/*w* formic acid), which provided the highest yield of 96.54%. For the production of calcium formate, 1 kg of CaCO_3_ (derived from shell waste) requires approximately 0.92 kg of pure formic acid. Considering the use of 60% (*w*/*w*) formic acid solution, the total required solution is approximately 1.53 kg, equivalent to 1.27 L based on a density of 1.205 kg·L^−1^. Under the optimal condition, 1 kg of CaCO_3_ can theoretically yield 1.30 kg of calcium formate, and when accounting for the experimental yield (96.54%), the actual product obtained is approximately 1.25 kg. The cost of shell-derived CaCO_3_ was estimated to be 0.033 USD·kg^−1^ [39], while the industrial bulk price of formic acid (85%) was approximately 0.68 USD·kg^−1^ (≈0.82 USD·L^−1^). Based on these values, the total raw material cost for producing 1.25 kg of calcium formate is approximately 1.07 USD, corresponding to a production cost of approximately 0.86 USD·kg^−1^. In addition to raw material costs, the operational expenses including plant, labor, water, and electricity were estimated to be approximately 0.030 USD·kg^−1^ [39], based on the previous cost evaluation framework used for calcium acetate monohydrate production. Therefore, the overall production cost of calcium formate in this work is approximately 0.89 USD·kg^−1^. To evaluate the economic competitiveness, this cost was compared with the market price of calcium formate in Thailand, which is approximately 1.09 USD·kg^−1^ (based on 25 kg bulk packaging). The result indicates that the proposed process remains economically viable, with a positive margin between production cost and market price. It should be noted that this cost estimation is preliminary and does not include additional expenses such as equipment depreciation, maintenance, and quality control. These factors may influence the overall production cost in industrial applications. In addition, variations in raw material prices and regional economic conditions should also be considered.

Overall, the proposed process demonstrates strong potential for practical applications due to its simplicity, rapid reaction, and cost-effectiveness. The direct reaction between cockle shell powder and concentrated formic acid eliminates complex processing steps and avoids the use of additional organic solvents for product precipitation [12]. Moreover, using shell waste as a renewable calcium source supports waste valorization and reduces environmental impacts associated with shell disposal and limestone mining. These advantages suggest that the developed method could serve as a promising and sustainable approach for producing calcium formate from seafood processing waste, with potential scalability for industrial applications in construction additives [16,17,18], feed supplements [19], and other chemical industries [20,21,22].

## 3. Materials and Methods

### 3.1. Precursors

Formic acid (85% *w*/*w*, Parchem, New Rochelle, NY, USA) was used as a precursor of the formate anion for the synthesis of calcium formate without further purification. Concentrated formic acid 85% *w*/*w* was diluted with de-ionized water to prepare 50%, 60%, and 70% *w*/*w* formic acid.

Cockle shells were collected from the Ang Sila fish market, Chonburi, Thailand. The collected shells were cleaned with sodium hypochlorite (5% *w*/*v*, NaOCl, Sigma-Aldrich, Saint Louis, MO, USA) solution and then dried in an oven at 105 °C for 1 h. The dried shells were ground and sieved through a 100-mesh sieve to obtain the fine shell powders (CaCO_3_). The CaCO_3_ precursor was analyzed for chemical composition and crystal phase using XRF and XRD, respectively. The result of the chemical composition of the CaCO_3_ precursor was found to be 94.7%CaO, 1.47%Na_2_O, 1.15%SiO_2_, 1.06%P_2_O_5_, 0.584%MgO, 0.550%Al_2_O_3_, 0.260% CuO, and 0.127%K_2_O, so the purity of this agent is about 94.7% [10].

### 3.2. Preparation of Calcium Formate (Ca(HCOO)_2_)

The prepared cockle shell powders (CK) and diluted formic acid (50%, 60%, and 70% *w*/*w*) were used as starting materials without further purification. For each experiment, formic acid (50%, 60%, or 70% *w*/*w*) was slowly added to a beaker containing 10 g of CK at a mole ratio of CK (CaCO_3_): Formic acid (HCOOH) = 1:2 according to Equation (3). At the same time, continue stirring at 400 rpm using a magnetic stirrer (IKA, Staufen, Germany). Finally, the mixed suspensions were dried until their weight no longer changed and then kept in the desiccator for characterization. All calcium formate preparations were prepared in triplicate to ensure the accuracy of the production parameters. The samples obtained were labeled as CF50, CF60, and CF70, corresponding to 50%, 60%, and 70% *w*/*w* formic acid, respectively.

CaCO_3_(s) + 2HCOOH(l) → Ca(HCOO)_2_(s) + H_2_O(l) + CO_2_(g) (3)

### 3.3. Characterization of Materials

The chemical composition and structure of CF50, CF60, and CF70 were characterized by scanning electron microscopy (SEM), X-ray diffraction (XRD), X-ray fluorescence (XRF), Fourier transform infrared (FTIR) spectroscopy, and thermogravimetric analysis (TGA). For SEM, the morphological evaluation of all prepared samples was carried out using a Leo SEM 400 scanning electron microscope (15 kV accelerating voltage, 5000×, SEM, VP1450, LEO, North Billerica, MA, USA) after coating the samples with gold. For XRD, the crystal structure of all samples was analyzed by the Rigaku MiniFlex X-ray diffractometer (MiniFlex, Rigaku Corporation, Tokyo, Japan) from 5° to 60° 2θ. For XRF, the chemical composition of all samples was examined by the Rigaku ZSX PrimusIV X-ray fluorescence (Rigaku Corporation). Samples were mixed with binding to produce a homogeneous sample pellet. For FTIR, the functional groups of all samples were identified using a PerkinElmer Spectrum GX FTIR spectrophotometer (Spectrum GX, PerkinElmer, Waltham, MA, USA) in the range of 400–4000 cm^−1^ with KBr pellets. For TGA, the thermal decomposition of samples was analyzed by the Perkin–Elmer Pyris Diamond thermogravimetric/differential thermal analyzer (TG-DTA) from 30 to 900 °C. A total of 30 µg of each sample was placed into the sample TG pan. The samples were analyzed under N_2_ atmosphere with a N_2_ flow rate of 20 mL·min^−1^ at a heating rate of 10 °C·min^−1^.

## 4. Conclusions

This study demonstrated a rapid and simple method for synthesizing calcium formate from cockle shell waste through the direct reaction of shell-derived calcium carbonate with high-concentration formic acid. The experimental results revealed that formic acid concentration plays a crucial role in determining reaction behavior and product quality. The samples prepared using 50% and 60% formic acid produced well-crystallized α-calcium formate with high phase purity, as confirmed by XRD and FTIR analyses. Among the investigated conditions, 60% formic acid was identified as the optimal synthesis condition because it provided complete conversion to calcium formate with the highest yield and the shortest synthesis time. In contrast, the reaction using 70% formic acid was highly exothermic, hindering complete conversion and leaving residual calcium carbonate in the final product. This incomplete reaction also decreased the solubility and yield of the synthesized calcium formate. The thermal decomposition of samples was similar to the previous works, which confirms that anhydrous calcium formate (Ca(HCOO)_2_) was synthesized. Overall, the developed process offers several advantages, including short synthesis time, simplified operation, reduced chemical usage, and lower production costs. In addition, the utilization of cockle shell waste as a renewable calcium source contributes to waste valorization, reduces environmental impacts associated with shell disposal, and decreases reliance on mined limestone resources, with excellent potential for application in industrial-scale material production.

## Figures and Tables

**Table 1:** Vibrational characteristics (mode) and vibrational positions (wavenumber) of all the synthesized calcium formate (Ca(HCOO)_2_); CF50, CF60 and CF70 samples.

Vibrational Modes	Wavenumber/cm^−1^	Assignment
O–H stretching of H_2_O	3711–3029	*ν*_as_(O–H)
C–H asymmetric stretching	2908–2842	*ν_a_*_s_(C–H)
C=O symmetric stretching	1682–1423	*ν*_s_(C=O)
O–C–H bending	1423–1363	*δ*(O–C–H)
C–O asymmetric stretching	1363–1266	*ν*_as_(C–O)
O=C–O bending	820–723	*δ*(O=C–O)
Ca–O	500–400	*ν*(Ca–O)

**Table 2:** Chemical composition and impurities of all the synthesized calcium formate (Ca(HCOO)_2_); CF50, CF60 and CF70 samples (wt. %).

Compounds	Formula	Chemical Contents/wt%		
		CF50	CF60	CF70
Calcium oxide	CaO	95.50	95.28	93.34
Disodium oxide	Na_2_O	0.96	0.93	1.80
Magnesium oxide	MgO	0.17	0.20	0.27
Aluminum oxide	Al_2_O_3_	0.55	0.58	0.75
Silicon dioxide	SiO_2_	1.33	1.40	1.80
Phosphorous oxide	P_2_O_5_	0.06	0.05	0.06
Sulfur oxide	SO_3_	0.10	0.10	0.18
Chlorine	Cl	0.01	0.01	0.02
Dipotassium oxide	K_2_O	0.02	0.02	0.03
Titanium dioxide	TiO_2_	-	0.01	0.16
Manganese oxide	MnO	0.05	0.04	0.07
Ferric oxide	Fe_2_O_3_	0.92	0.99	0.99
Strontium oxide	SrO	0.33	0.30	0.37
**Total**		**100.00**	**100.00**	**100.00**

**Table 3:** The production parameters of all the synthesized calcium formate (Ca(HCOO)_2_); CF50, CF60, and CF70 samples from cockle shells using 50%, 60%, and 70% *w*/*w* formic acid.

Samples	Formic Acid Concentration /% *w*/*w*	Reaction Temperature /°C	Reaction Time/min	Yield/%	Soluble Percentage/%
CF50	50	44 ± 3	20 ± 4	95.88 ± 1.25	93.87 ± 1.32
CF60	60	49 ± 2	15 ± 2	96.54 ± 1.47	94.49 ± 1.13
CF70	70	67 ± 4	1 ± 1	88.69 ± 1.78	63.14 ± 2.03

## Data Availability

The original contributions presented in this study are included in the article. Further inquiries can be directed to the corresponding author.
